# Protocol for a systematic review and meta-analysis on the clinical outcomes and cost of deep inferior epigastric perforator (DIEP) flap versus implants for breast reconstruction

**DOI:** 10.1186/s13643-017-0628-y

**Published:** 2017-11-22

**Authors:** Ankur Khajuria, Oliver J. Smith, Maxim Prokopenko, Maximillian Greenfield, Afshin Mosahebi

**Affiliations:** 1 Department of Surgery and Cancer, Imperial College London, London, UK; 2 Department of Plastic & Reconstructive Surgery, Royal Free Hospital NHS Trust, London, UK

**Keywords:** Breast implant, DIEP, Cost-effectiveness, Autologous flap reconstruction, Deep inferior epigastric artery perforator flap

## Abstract

**Background:**

Mastectomy in the context of breast malignancy can have a profoundly negative impact on a woman’s self-image, impairing personal, sexual and social relationships. The deep inferior epigastric perforator (DIEP) flap and implants are the two commonest reconstructive modalities that can potentially overcome this psychological trauma. The comparative data on clinical outcomes and costs of the two modalities is limited. We aim to synthesise the current evidence on DIEP versus implants to establish which is the superior technique for breast reconstruction, in terms of clinical outcomes and cost-effectiveness.

**Methods:**

A comprehensive search will be undertaken of EMBASE, MEDLINE, Google Scholar, CENTRAL and Science citation index databases (1994 up to August 2017) to identify studies relevant for the review. Primary human studies evaluating clinical outcomes and cost of DIEP and implant-based reconstruction in context of breast malignancy will be included. Primary outcomes will be patient satisfaction and cosmetic outcome from patient-reported outcome measures (scores from validated tools, e.g. BREAST-Q tool), complications and cost-analysis. The secondary outcomes will be duration of surgery, number of surgical revisions, length of stay, availability of procedures and total number of clinic visits.

**Discussion:**

This will be the first systematic review and meta-analysis in available literature comparing the clinical outcomes and cost-effectiveness of DIEP and implants for breast reconstruction. This review is expected to guide worldwide clinical practice for breast reconstruction.

**Systematic review registration:**

PROSPERO CRD42017072557.

**Electronic supplementary material:**

The online version of this article (10.1186/s13643-017-0628-y) contains supplementary material, which is available to authorized users.

## Background

Breast cancer is the commonest malignancy in women and a major cause of cancer-related mortality [1]. While mastectomy is the primary treatment modality for these patients, it can have a profoundly negative impact on their lives, impairing personal, sexual and social relationships. Fifty percent of women post-mastectomy suffer from negative self-image with negative changes in their sexuality [2, 3]. The demand for reconstructive procedures has risen, not only as a consequence of advancing cancer treatment but also because of the demonstrated functional, psychological and social benefits for patients, overcoming the psychological trauma associated with mastectomy [4–9]. The rates of post-mastectomy breast reconstruction doubled from 13 to 26% between 1998 and 2007 [10].

A number of reconstructive techniques exist for breast reconstruction. The two most frequently employed techniques include the autologous deep inferior epigastric perforator (DIEP) flap and implant-based reconstruction [11]. The choice of treatment is determined by combination of patient factors (individual preference, age, body image) and surgeon factors (team experience, availability of resources) [8, 9]. Despite this, many plastic surgery units worldwide regard autologous flap reconstruction as the superior technique as it follows the paradigm of replacing ‘like with like’ [10]. Indeed, there is growing evidence to support increased aesthetic patient satisfaction with autologous flap reconstruction compared to implants, as well as increased suppleness and resiliency, especially in irradiated recipient beds [11–19].

On first glance, implant-based reconstruction is a simpler technique compared to free flap reconstruction, requires less training and time and can be performed by many more surgeons [15]. However, implant-based reconstruction has complications. These include migration, implant rupture, infection, exposure/extrusion and patient dissatisfaction with edge visibility and implant animation [20]. Capsular contracture can result in pain, asymmetry, increased palpability and requirement of implant removal [21]. The placement of the implant itself can lead to reduced or absent sensation at the nipple in 1 in 7 women [20]. Allergan’s 10-year cumulative risk study found that 24.6% of patients who underwent implant-based reconstruction developed capsular contracture necessitating implant removal and/or replacement [22].

Conversely, DIEP flap is often now considered the gold-standard autologous flap reconstructive technique. This is because it results in less abdominal donor site morbidity compared to the traditional transverse rectus abdominus myocutaneous (TRAM) flap, by preserving the continuity of the rectum abdominis muscles [23]. Compared to implant-based reconstruction, some authors have argued that DIEP flap reconstruction is more cost-effective and results in fewer complications [11, 24]. Modern healthcare aims to provide cost-effective treatment, and thus, discussion on reconstructive modalities warrants scrutiny on cost associated with autologous and implant-based reconstruction. While some North American and European centres have published cost-effectiveness analysis on DIEP versus implants, the data is sparse and there is a relative scarcity of inclusion of data from public and free universal healthcare system settings [11].

Thus, an extensive search will be undertaken in the MEDLINE (Ovid SP), EMBASE (OvidSP), Google Scholar, Cochrane Central Register of Controlled Trials and Scientific Citation Index databases to identify primary studies on DIEP (intervention) and implant-based reconstruction (comparator) in context of patients with breast malignancy. Data extracted will be used to evaluate which technique is superior in terms of clinical outcomes and cost and thus inform worldwide clinical practice.

## Methods

### Objective

This systematic review is intended to evaluate the current evidence on the clinical outcomes and cost of deep inferior epigastric perforator (DIEP) flap versus implants for breast reconstruction post cancer-related mastectomy, to determine which technique is more cost-effective and clinically superior.

### General methods

This protocol has been registered with the National Institute of Health Research (NIHR) Prospective Register of Systematic Reviews PROSPERO CRD42017072557. We have adhered to and completed the Preferred Reporting Items for Systematic Review and Meta-Analysis Protocols (PRISMA-P) 2015 statement [25] (please see Additional file 1, PRISMA-P checklist). If no randomised controlled trials (RCT) are available, the review will be reported according to the Meta-Analysis of Observational Studies in Epidemiology (MOOSE) guidelines [26].

### Search strategies

We will conduct a comprehensive search of the MEDLINE (OVID SP), EMBASE (OVID SP), Google Scholar, CENTRAL and Science citation index databases from 1994 up to August 2017 to identify studies relevant to the review. A combination of Medical Subject Headings (MeSH) terms and free text will be used, combined with Boolean logical operators to construct the search strategy. Explode function will be used to capture narrower terms. No language or study design restrictions will be applied. The reference list of all included articles will also be screened for relevance. A sample search strategy, for EMBASE (OVID SP), is shown below and a similar strategy will be adapted for the other databases:

- (1) exp Breast Neoplasms/ OR ((breast adj6 cancer*) or (breast adj6 neoplasm*) or (breast adj6 carcinoma*) or (breast adj6 tumour*) or (breast adj6 tumor*) or (breast* adj4 reconstruct*))
- (2) exp deep inferior epigastric perforator flap/ OR DIEP flap* OR DIEAP flap* OR ((Deep and inferior and epigastric and perforator) adj2 flap*) OR Deep and inferior and epigastric and perforator and flap*)
- (3) exp breast implant/ OR breast adj3 implant* OR exp silicone prosthesis/
- [(1) AND (2)] OR [(1) AND (3)]; publication date: January 1994–August 2017

### Identification and selection of studies

Studies extracted following database searching will be populated into an Endnote X7 library (Clarivate Analytics, USA). The screening will be carried out in two stages using pre-specified screening criteria by two independent reviewers. Inter-rater reliability will be calculated using Cohen’s kappa score.

- Stage 1: Title and abstract screening will be carried out by two researchers acting independently. Any discrepancies will be resolved by consensus. If any doubt remains, the article would usually proceed to full-text review.
- Stage 2: The full-text versions of the studies included in Stage 1 will be downloaded and screened for eligibility by two researchers independently. Discrepancies will again be resolved by consensus. If this is not possible, a third author will be consulted.

### Study design

All primary human studies evaluating clinical outcomes and cost of DIEP and implant-based reconstruction in context of breast malignancy will be included. The intervention is the DIEP flap and the comparator is implant-based reconstruction. The inclusion and exclusion criteria are highlighted below.

#### Inclusion criteria

a Studies involving adult patients between 18 and 90 years old.
b Unilateral DIEP flap or implant-based breast reconstruction due to breast cancer (bilateral reconstruction is usually after bilateral prophylactic mastectomy).
c Clinical studies (randomised controlled trials, prospective and retrospectives cohort studies, case series).

#### Exclusion criteria

- Review articles, case reports, simulation studies, clinical studies in non-human subjects, patients with segmental or partial mastectomy, technical descriptions of operative repair with no outcome measures, breast reconstruction not related to cancer, other autologous flap techniques.
- Duplicates will be excluded and studies will be screened for bias. The Cochrane’s risk of bias tool will be used for randomised controlled trials [27]. Bias will be assessed and judged as being high, low or unclear for individual elements from five domains (selection, performance, attrition, reporting and other) [27]. For non-randomised comparative studies, ROBINS-I (Risk Of Bias In Non-randomised Studies—of Interventions) by Cochrane will be used [28]. ROBINS-I covers seven distinct domains from which bias may be introduced, with ‘signalling questions’ that facilitate judgements about the risk of bias. The judgements within each domain will be carried forward for an overall risk of bias judgement across bias domains [28]. Studies affected by bias will be excluded.

### Outcomes

The primary outcomes will be:

1. Patient satisfaction and cosmetic outcome from patient-reported outcome measures (PROMs, scores from validated tools, e.g. BREAST-Q tool)
2. Complications (arterial thrombosis, fat necrosis, venous congestion, infection, partial/full flap loss, donor site complications, haematoma/seroma, return to theatre, capsular contracture, scarring, implant deflation/rupture/displacement)
3. Cost-analysis

The secondary outcomes will be:

1. Duration of surgery
2. Number of surgical revisions
3. Length of stay
4. Availability of procedures
5. Total number of clinic visits

If the data is appropriate for quantitative synthesis, then risk ratio with 95% confidence interval (CI) will be used to determine dichotomous outcomes (complications). Continuous outcomes (cost, PROMs [BREAST-Q], secondary outcomes excluding availability of procedures) will be determined by weighted or standardised mean differences with 95% CI. Subgroup analysis may be performed for patients with different breast cancer types and for breast implant materials, dependent on sufficient data sets. Where possible, we will utilise results from an intention to treat analysis.

### Data extraction, collection and management

Data, from the full-text articles, will be extracted by two independent authors using a standardised extraction form. Any discrepancy will be resolved by consensus or with referral to a third author. If any data is missing or further information is required, the primary authors of the manuscript will be contacted directly. The following data will be extracted:

- first author
- year of publication
- study design
- study setting
- study population
- participant demographics (sex, mean age, BMI, comorbidity)
- complications (arterial thrombosis, venous congestion, infection, fat necrosis, partial/full flap loss, haematoma/seroma, donor site complications, return to theatre, capsular contracture, scarring, implant, deflation/rupture/displacement)
- measures of patient satisfaction (PROMs e.g. BREAST-Q)
- economic data

An assessment of heterogeneity will be performed using Review Manager 5.3 provided by The Cochrane Collaboration [29]; if the studies are relatively homogenous in terms of methodology and outcomes, meta-analyses of the data will be performed. If there is high heterogeneity, a narrative synthesis will be performed instead, without meta-analysis.

Statistical heterogeneity will be quantified by the *I* ^2^ statistic [30]. If the *I* ^2^ statistic is high, indicating high heterogeneity, a random effects model will be employed. The Grading of Recommendations Assessment, Development and Evaluation (GRADE) approach [31] will be utilised to assess the methodological quality of the studies. Cochrane has produced GRADE tables that identify the basis for judgements about evidence quality. An overall GRADE score (from 4 to 0) is calculated based on quality of overall evidence. The tables specify why points may be added or deducted to obtain the final score [31]. Sensitivity analysis maybe performed based on the quality of the studies, with analyses repeated after removal of poor quality studies to evaluate any change in the overall effect estimate.

## Discussion

The aim of this review is to evaluate the clinical outcomes and cost of DIEP flap versus implants for breast reconstruction in context of breast malignancy. Despite many centres ascribing DIEP flap as the gold-standard reconstructive modality, data on clinical outcomes and cost-effectiveness is limited. Therefore, it is important to determine which of the two techniques is clinically superior and more cost-effective as this will guide clinical management. To our knowledge, this is the first systematic review to compare the clinical outcomes and cost of DIEP versus implants.

### Dissemination

Based on the results of this systematic review, independent recommendations will be made to plastic surgeons, researchers, policy makers and plastic surgery societies. The results will be disseminated at international meetings in the fields of Plastic, Reconstructive and Aesthetic Surgery and published in a high-impact peer-reviewed journal.

## Additional file

Additional file 1: PRISMA-P checklist. (DOCX 509 kb)

## Abbreviation

CENTRAL: Cochrane Central Register of Controlled Trials
DIEP: Deep inferior epigastric perforator
EMBASE: Excerpta Medica Database
GRADE: Grading of Recommendations Assessment, Development and Evaluation
MeSH: Medical Subject Headings
MOOSE: Meta-Analysis of Observational Studies in Epidemiology
PRISMA-P: Preferred Reporting Items for Systematic Review and Meta-Analysis Protocols
RCT: Randomised controlled trial
TRAM: Transverse rectus abdominus myocutaneous
